# CD44 expressed by myeloid cells promotes glioma invasion

**DOI:** 10.3389/fonc.2022.969787

**Published:** 2022-08-04

**Authors:** Ekaterina L. Ivanova, Barbara Costa, Tanja Eisemann, Sabrina Lohr, Pavle Boskovic, Viktoria Eichwald, Jasmin Meckler, Manfred Jugold, Veronique Orian-Rousseau, Heike Peterziel, Peter Angel

**Affiliations:** ^1^ Division of Signal Transduction and Growth Control, DKFZ/ZMBH Alliance, German Cancer Research Center (DKFZ), Heidelberg, Germany; ^2^ Division of Molecular Genetics, German Cancer Research Center (DKFZ), Heidelberg, Germany; ^3^ Core Facility Small Animal Imaging Center, German Cancer Research Center (DKFZ), Heidelberg, Germany; ^4^ Karlsruhe Institute of Technology (KIT), Institute of Biological and Chemical Systems – Functional Molecular Systems (IBCS-FMS), Hermann-von-Helmholtz-Platz 1, Eggenstein-Leopoldshafen, Germany; ^5^ Clinical Cooperation Unit Pediatric Oncology, German Cancer Research Center (DKFZ), Heidelberg, Germany

**Keywords:** glioblastoma, CD44, tumor microenvironment, microglia, TLR2, MMP9

## Abstract

Glioblastoma multiforme (GBM) is one of the most common and malignant brain tumors in adulthood with a median survival of only 15 months. This poor prognosis is related to GBM’s ability to extensively infiltrate the surrounding brain parenchyma resulting in diffuse spread of neoplastic cells in the brain, responsible for high rate of recurrence. CD44 (Cluster of Differentiation 44) is a transmembrane protein, overexpressed in multiple cancer types, including gliomas, and implicated in cell motility, proliferation and angiogenesis. Multiple studies have investigated the role of CD44 in GBM cells and have highlighted a link between tumor malignancy and CD44 expression. However up to date, little is known of the role of CD44 on cells from the tumor microenvironment (TME). Here, we have investigated a potential role of CD44 in the TME in regards to GBM invasiveness. Using an ex-vivo organotypic brain slice invasion assay, we show that absence of CD44 from the TME impairs the ability of glioma cells to invade the surrounding brain parenchyma. By deleting CD44 in the astrocytic, endothelial and myeloid compartments, we show that it is specifically CD44 expression in myeloid cells that is responsible for the observed phenotype. Combining *in vivo* studies in cell-specific knock-out mice and *in vitro* analyses on primary microglia we demonstrate that myeloid CD44 is implicated in Toll Like Receptor 2 signaling and is a major regulator of Matrix metalloproteinase 9 expression.

## Introduction

Glioblastoma multiforme (GBM) is one of the most common malignant brain tumors in adulthood. Despite intensive research and therapy development, the median survival time is currently only 15 months and the 5-year survival rate is less than 3% (1, 2). This poor prognosis is related to the diffuse nature of the disease. There is urgent need for more efficient therapeutic options whose development however requires a deeper understanding of glioblastoma invasiveness. Many studies focused on this subject and have highlighted the complexity in the mechanisms and signaling pathways implicated, as well as the pivotal role of the tumor microenvironment (TME) [reviewed in (3, 4)].

Cluster of differentiation CD44 (CD44) is a transmembrane glycoprotein involved in cell-cell and cell-matrix interactions, expressed ubiquitously by the majority of human tissues, such as brain, endocrine and respiratory system, digestive tract, liver, kidney and bone marrow (5). The main ligand of CD44 is hyaluronic acid (HA), a glycosaminoglycan and a chief component of the extracellular matrix (ECM) (6). Other major ECM CD44 ligands are osteopontin, collagens, integrins, laminin, fibronectin and matrix metalloproteinases (MMPs) (5, 7). CD44 additionally functions as a co-receptor for a plethora of growth factors and cytokines by interacting, among others, with receptor tyrosine kinases (RTKs) and G protein-coupled receptors (GPCRs) (8, 9).

CD44 is overexpressed in multiple cancer types such as pancreatic, salivary gland, head and neck, leukemia/lymphoma [reviewed in (10–13)], and is one of the most common cancer stem cell surface marker (7, 14, 15). In glioma, CD44 abundance correlates with tumor grade (16–18) and predicts poor survival rates (19). CD44 is not only expressed by cancer cells, particularly cancer stem cells, but also in cell types from the glioblastoma TME, including high-grade GBM-associated astrocytes (16, 20), endothelial cells (21, 22), resident and infiltrating immune cells like microglia, macrophages and T lymphocytes (18). However, up to date little is known about CD44’s role in these cells in regard to glioma progression.

Considering its increased levels correlating with glioma grade and malignancy and given its broad expression pattern within the TME, we considered CD44 to be an interesting target to investigate in the context of tumor-host interactions affecting glioma invasion. Here, we demonstrate that myeloid-specific but neither astrocyte- nor endothelial-specific CD44 deletion significantly affected glioma invasiveness in organotypic brain slices. In light of its described role in Toll Like Receptor 2 (TLR2) signaling, we studied the implication of CD44 in myeloid-derived pro-inflammatory cytokines and MMPs production and found that CD44 affects mRNA levels of TNF-α, IL-1b and MMP9 both *in vivo* and *in vitro*.

## Results

### CD44 in the TME is required for spheroid invasion on brain slices

**Figure 1:**
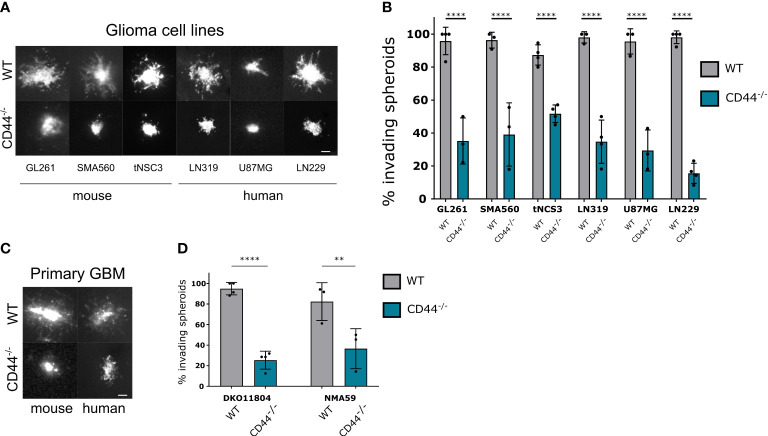
CD44 in the TME is required for glioma cell migration. **(A, B)** Mouse and human established glioma cell lines were assessed for their ability to invade in the absence of CD44 from the TME. Ex-vivo cultured brain slices were prepared from WT and CD44^-/-^ mice. GL261, SMA560, tNSC3 mouse glioma cells and LN319, U87MG, LN229 human glioma cells were seeded in an 1% low melting agar-coated 96-well plate and allowed to form spheroids, which were then implanted into the brain slices. **(A)** Representative images of DiD (lipophilic carbocyanine dye) labeled glioma spheroids 48 hours after implantation, scale bar 100μm. **(B)** The number of invading spheroids was counted for each cell line and presented as percentage of total counted spheroids. Bar charts represent the percentage of invading spheroids from at least 3 experiments. An average of 10-20 spheroids were evaluated per experiment. Two-way ANOVA with Sidak’s multiple comparisons test, ****p < 0.0001. **(C, D)** Spheroids of mouse (DKO11804) of human (NMA59) primary glioma cells were implanted into organotypic brain slices from WT and CD44^-/-^ mice. **(C)** Representative images of DiD labeled glioma spheroids 48 hours after implantation, scale bar 100μm. **(D)** Quantification of the number of invading spheroids (n≥3 experiments, 10-20 spheroids per experiment). Bar charts represent the relative percentage of invading spheroids for each cell line and mouse genotype. Two-way ANOVA with Sidak’s multiple comparisons test, **p < 0.01; ****p < 0.0001.

To investigate the impact of CD44 in the TME on tumor invasion we used the approach of organotypic brain slices (23). We prepared brain slices from mice with germinal deletion of CD44, hereafter CD44^-/-^ (24), versus wild-type (WT) mice and implanted them with spheroids from different mouse or human glioma cell lines labelled with a fluorescent dye. Forty-eight hours past implantation, the majority of spheroids implanted in WT brain slices presented an invasive behavior and spread of cells could be observed around the initial spheroid mass (**Figure 1A**). However, on slices from CD44^-/-^ mice, this phenotype could be detected only for a limited number of spheroids and for various glioma cell lines we observed disruption in invasiveness (**Figure 1B**). Similar results were obtained for primary glioma cells from mouse (DKO11804 (25); or human (NMA59) tumors (**Figures 1C, D**), demonstrating that absence of CD44 from cells of the glioma microenvironment disrupts invasion.

### CD44 on myeloid cells modulates glioma invasion

**Figure 2:**
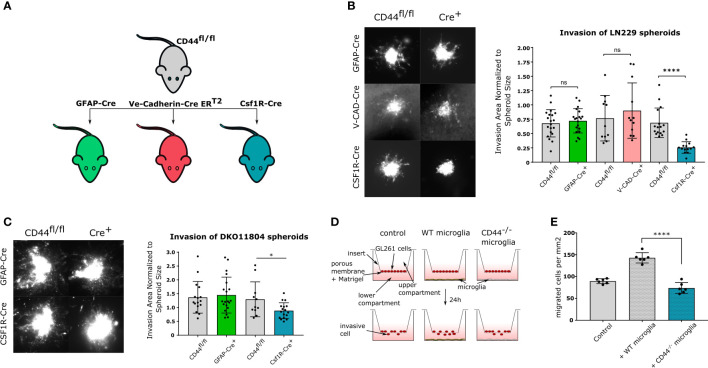
CD44 in myeloid cells stimulates glioma invasion **(A)** Schematic representation of mouse lines used in the study. CD44^fl/fl^ mice were crossed with three different Cre-bearing mouse lines in order to generate conditional knock-out models. In CD44^fl/fl^ × GFAP-Cre mice, CD44 is specifically deleted in astrocytes; in CD44^fl/fl^ × Ve-Cadherin CreER^T2^ mice, upon tamoxifen treatment CD44 is specifically deleted in endothelial cells; and in CD44^fl/fl^ × Csf1R-Cre mice, CD44 is specifically deleted in myeloid cells. **(B)** Ex-vivo invasion assay on LN229 spheroids implanted in brain slices from the three cKO mouse models. CD44^fl/fl^ mice were used as controls. Representative images of DiD labeled LN229 spheroids 48 hours after implantation are shown on the left. Corresponding quantification is shown on the right. Bar charts represent the invasion area of a spheroid normalized to the area of its mass. Data are presented as mean ± SD. Student’s t-Test, ****p < 0.0001; n.s. = not-significant. **(C)** Ex-vivo invasion assay on DKO11804 primary mouse glioma cells implanted in brain slices from CD44^fl/fl,^ CD44^fl/fl^×GFAP-Cre and CD44^fl/fl^×Csf1R-Cre mice. Representative images of DiD labeled DKO11804 spheroids 48 hours after implantation are shown on the left. Corresponding quantification is shown on the right. Bar charts represent the invasion area of a spheroid normalized to the area of its mass. Data are presented as mean ± SD. Student’s t-Test, *p < 0.05. **(D)** Schematic representation of the Boyden assay. **(E)** Quantification of migrating GL261 cells. Bar charts represent the number of transmigrating cells per mm^2^. One-way ANOVA with the Tukey’s multiple comparisons test, ****p < 0.0001.

We next sought to define the cell type within the TME responsible for CD44-mediated invasiveness. To this aim, we generated three conditional mouse lines, by crossing CD44^fl/fl^ mice (26) with GFAP-Cre (27), VE-Cadherin-Cre ER^T2^ (28) or CSF1R-Cre (29) mice to specifically delete CD44 in astrocytes, endothelial cells or myeloid cells, respectively (**Figure 2A**). Immunofluorescence analysis on isolated astrocytes, endothelial cells, microglia and macrophages from these lines confirmed successful and specific CD44 knock-out in the targeted cells (**Supplementary Figures 1A–D**). Invasion was evaluated by the organotypic brain slices assay using the LN229 cell line, which presented the strongest phenotype in our initial assay. By dividing the invasion area of spheroid cells to spheroid size, we found that CD44 deletion in either astrocytes or in endothelial cells does not affect glioma migration (**Figure 2B**). On the contrary, myeloid-specific deletion of CD44 significantly reduces invasiveness, strongly suggesting that CD44 expression in myeloid cells of the TME promotes glioma invasion. We confirmed these observations with primary glioma DKO11804 cells (**Figure 2C**). Invasion was again impaired in the context of myeloid-CD44 deletion, but not upon astrocyte-specific deletion of CD44.

To test whether the observed invasion-facilitating effect of CD44 requires a direct contact between myeloid and tumor cells, we employed the Boyden chamber protocol using transwell units coated with Matrigel, a polymer mix mimicking the extracellular matrix. Primary microglia isolated from WT or CD44^-/-^ mice were seeded in the bottom compartment, while adherently growing GL261 mouse glioma cells were seeded in the top compartment and their migration was assessed after 24 hours (**Figure 2D**). In the absence of microglia, an average of 86.56 ( ± 5.66) GL261 cells per mm^2^ migrated through the Matrigel (**Figure 2E** left). When WT microglia were added, this number drastically increased to 142.9 ( ± 11.78). In contrast, no increase was observed in the presence of CD44^-/-^ microglia (73.91 ± 12.42) (**Figure 2E** right) confirming that the tumor invasion promoting effect of myeloid cells is CD44-dependent. Given the experimental setting using transwell units, we hypothesize that glioma invasion-promoting factors secreted by microglia are not produced or released in the absence of CD44.

### Effects on GBM invasion and tumor volume *in vivo*

**Figure 3:**
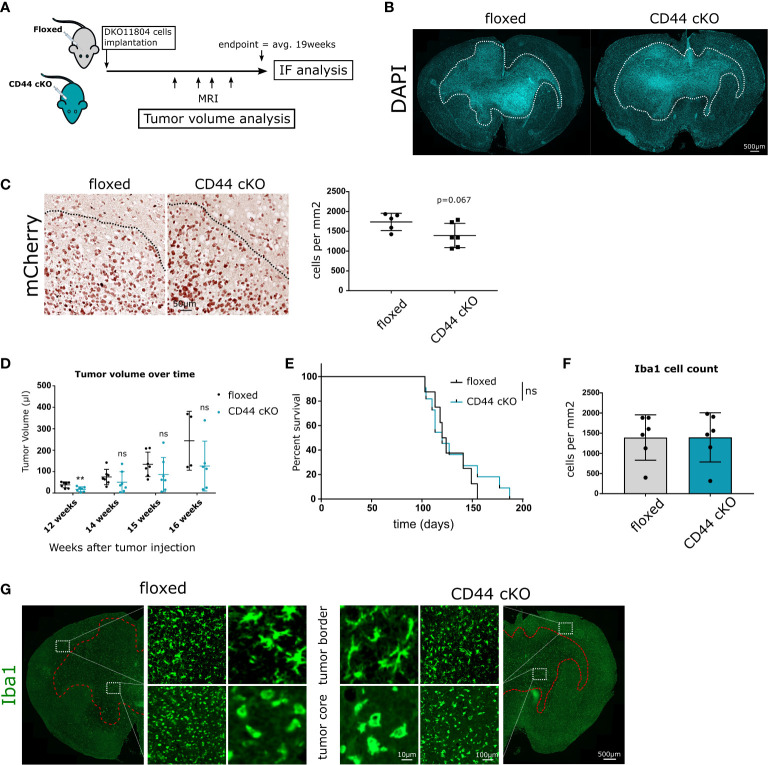
Effects of Myeloid-specific CD44 knock-out *in vivo*. **(A)** 1 × 10^6^ DKO11804 mCherry labelled cells were implanted in 5-8 weeks old floxed or CD44 cKO mice. MRI imaging was performed at weeks 12, 14, 15 and 16 after tumor cells implantation. Animals were sacrificed at endpoint, on average 19 weeks after injection and IF/IHC Analysis were performed. **(B)** Representative images of brains from floxed and CD44 cKO tumor-bearing mice stained with DAPI. Dashed line delimits tumor and shows invasive growth pattern. **(C)** Left-Representative images of tumors stained with mCherry. Dashed lines delimit the tumor core. Right-Quantification of the number of mCherry-positive tumor cells migrating beyond the tumor border. The tumor border was manually drawn and the number of cancer mCherry-positive cells spreading in a radius of 300 microns was counted. Data are presented as mean ± SD. Student’s t-Test, p=0.067. **(D)** Tumor growth was monitored with MRI. Graphs represent tumor volume (μl) for individual mice calculate from T2- weighted images.” by “Graphs represent tumor volume (μl) for individual mice calculated from T2-weighted images. Data are presented as mean ± SD. Student’s t-Test, ** p < 0.01; n.s. = not-significant. **(E)** Kaplan-Meier plot of survival for floxed (n=11) and cKO (n=8) mice transplanted with 1 × 10^6^ DKO11804. Time = days. Log-rank (Mantel-Cox) test, n.s. = not-significant. **(F–G)** Myeloid cells recruitment and morphology at endpoint. **(F)** Bar charts depict the number of Iba1 positive cells per mm^2^ in the tumor core. Student’s t-Test. **(G)** Representative images of Iba1- positive cells. At the tumor border, labelled cells present with ramified morphology, whereas within the tumor core their morphology is amoeboid.

To study the impact of CD44-positive myeloid cells on glioma growth and progression *in vivo*, we carried out intracranial orthotopic injections in CD44^fl/fl^ (floxed) and Csf1r-Cre/CD44^fl/fl^ conditional knockout (cKO) mice. Analysis on glioma-associated myeloid cells (GAMs) isolated from control and cKO mice showed an average of 80% decrease in CD44 mRNA (**Supplementary Figure 1E**). Mice were injected with DKO11804 cells labelled with mCherry and were allowed to reach the end stage of tumor growth (approximately 19 weeks) at which point brains were collected for examination (**Figure 3A**). Immunohistological analysis revealed, that tumors had spread to the contralateral hemisphere and presented with an invasive and irregular shape in both genotypes (**Figure 3B**). To evaluate invasiveness, we first delimited the border of the tumor mass with the help of DAPI staining (**Figure 3B**).We then counted the number of mCherry labelled glioma cells that had spread in a perimeter of 300 μm beyond this border. Quantification showed on average fewer invading cells in cKO mice, although this did not reach significance (**Figure 3C**). Since DKO11804 cells exhibit an invasive growth pattern making the delimitation of the tumor zone fairly delicate, we repeated the experiment with GL261 cells, which grow very aggressively in a much bulkier fashion, with a clearly discernible tumor zone (**Supplementary Figures 2A, B**). In light of the much faster kinetics of tumor development upon injection of GL261 cells tumor cell invasion was determined already two weeks post-transplantation. The mean number of cells invading beyond the tumor border was again lower for cKO than for floxed mice, however, did not reach significance (**Supplementary Figure 2C**).

Monitoring tumor volume over time during tumor progression in mice injected with DKO11804 cells showed that for each individual time point, mean tumor volume in cKO mice is lower than in floxed mice (**Figure 3D**). Decreased tumor volume in cKO mice was significant at the 12 weeks after implantation time point, suggesting slower growth during early tumor stage. However, no prolonged survival of tumor-bearing mice harboring CD44 deletion in myeloid cells was observed (**Figure 3E**). Interestingly, injection of GL261 cells revealed a trend towards an even higher tumor volume in CD44 cKO mice two weeks post-injection (**Supplementary Figure 2D**). Most likely, due to the cell- intrinsic very high tumor growth rate of these cells, an effect of the TME cannot be seen within such a short time period. These data suggest that the strong phenotype in tumor cell migration observed in the *in vitro* brain slice cultures is not prominently manifested *in vivo* but only visible by reduced tumor volume during tumor progression and a trend in CD44-dependent reduction of tumor cell invasion upon CD44 deletion.

### CD44 does not affect myeloid recruitment in GBM

During gliomagenesis, neoplastic cells were shown to recruit brain resident and peripheral myeloid cells. This is paralleled by the polarization of myeloid cells towards a tumor-promoting phenotype (30). During this process, myeloid cells change their shape as well as molecular and functional properties (31). To investigate whether CD44 is implicated in myeloid cell recruitment and initial morphology shift we stained the collected brains for expression of the microglial and macrophage-specific marker Iba1 (Ionized calcium binding adaptor molecule 1). No differences were observed in the number of Iba1-positive cells within the tumor between floxed and cKO animals (**Figure 3F** and **Supplementary Figure 2E**). Additionally, we investigated the morphology of Iba1-positive cells in the tumor core and in the vicinity of the tumor border. In both, floxed and cKO mice, myeloid cells in the tumor core presented with an amoeboid morphology (**Figure 3H** and **Supplementary Figure 2F**), characteristic for an activated state (31). Myeloid cells at the tumor border presented with a ramified morphology corresponding to a surveillance mode. These data imply that lack of CD44 in myeloid cells affects neither their recruitment to the tumor, nor their initial morphological response.

### Microglial CD44 is implicated in TLR2 signaling

Following their active recruitment to the tumor and their protumorigenic activation, GAMs produce a large number of cytokines, extracellular matrix proteases and growth factors, promoting cancer growth and invasion. Interestingly, several reports have linked CD44 on monocytes and macrophages to pro-inflammatory cytokine production and release (32, 33). Later studies have added an additional element to this system, namely Toll-like receptor 2 (TLR2) by showing that TLR2 activation is responsible for pro-inflammatory TNF-α and IL-1b cytokine production and this process is dependent on CD44 (34, 35).

**Figure 4:**
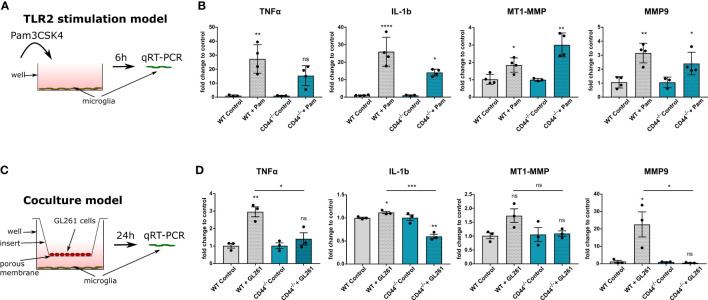
CD44 on myeloid cells is implicated in TLR2 signaling and MMP9 expression. **(A)** Schematic representation of the experimental setup. Primary microglia isolated from control or CD44^-/-^ mice were stimulated with 10ng/ml of the TLR2 agonist Pam3CSK4. After 6 hours, total RNA was extracted from the cells and processed for qRT-PCR analysis. **(B)** mRNA levels of TNF-α, IL-1b, MT1-MMP and MMP9 after 6 hours of Pam incubation. Bar charts represent mRNA levels as fold change to the corresponding control condition. Data are presented as mean ± SD. Student’s t-Test, ****p < 0.0001, **p < 0.01, *p < 0.05 vs corresponding control. **(C)** Schematic representation of the co-culture experimental setup. Primary microglia isolated from control or CD44^-/-^ mice were cultured in 6-well plates. GL261 cells were seeded in the top compartment on 0.4 µm porous inserts. For control condition, no cells were seeded on the insert. Co-culture was then carried-out for 24 hours after which total RNA was extracted from microglia and processed for qRT-PCR analysis. **(D)** mRNA levels of TNF-α, IL-1b, MT1-MMP and MMP9. Bar charts represent mRNA levels as fold change to the corresponding control condition. Data are presented as mean ± SD. Student’s t-Test, **p < 0.01, *p < 0.05 vs corresponding control. n.s., not-significant.

To investigate TLR2 signaling, we stimulated cultured primary microglia from control and CD44^-/-^ mice with the TLR2 agonist Pam3CSK4 (Pam condition) and evaluated mRNA levels of TNF-α and IL-1b (**Figure 4A**). Primary microglia isolated from both genotypes were evaluated for their purity and CD44 expression by IF staining (**Supplementary Figure 3A**). In microglia from control mice, TLR2 stimulation led to significantly increased levels of both TNF-α (by 27 times) and IL-1b (by 26 times) compared to untreated controls. For microglia from CD44^-/-^ mice, those levels still increased but to a much lesser extent, only by 15 and 14 times, respectively (**Figure 4B**). A direct comparison of the levels between the Pam condition for WT and the CD44^-/-^ microglia revealed levels 4 to 5 times lower in CD44^-/-^ microglia (**Supplementary Figure 3B**). Thus, similarly to macrophages, CD44 on microglia cells is implicated in pro-inflammatory cytokine production following TLR2 activation. Interestingly, CD44 levels in WT microglia were significantly increased upon Pam stimulation (**Supplementary Figure 3C**), further corroborating an interplay between CD44 and the TLR2 pathway.

Since TLR2 signaling in microglia has additionally been shown to regulate matrix metalloproteinases MMP9 and MT1-MMP (36, 37), we next evaluated their levels in our TLR2-stimulation model. Application of the Pam3CSK4 molecule led to significant increase in MT1-MMP levels for both WT and CD44^-/-^ microglia (**Figure 4B**). Unexpectedly, the increase was more prominent in CD44^-/-^ microglia (**Supplementary Figure 3B**). For MMP9, levels were increased 3.2 fold in WT and 2.4 fold in CD44^-/-^ microglia upon stimulation. A direct comparison between the Pam conditions revealed significantly lower levels of MMP9 for the CD44^-/-^ condition (**Supplementary Figure 3B**). Thus, upon direct TLR2 activation, CD44 knock-out in microglia appears to disrupt MMP9 upregulation, but facilitates MT1-MMP increase.

### Glioma cells trigger a CD44-dependent increase in TNF-α, IL-1b and MMP9 *in vitro*

In order to obtain insight into which kind of microglial secreted factors depend on CD44 in the context of glioma, we used a co-culture model in which primary microglia were incubated together with GL261 cells for 24h (**Figure 4C**). As control, no cells were seeded in the top compartment. Co-culture with GL261 cells increased TLR2 levels in WT microglia (**Supplementary Figure 3D**), consistent with TLR2 activation (36), but failed to do so in CD44^-/-^ microglia. Further qRT-PCR analysis revealed that in the presence of glioma cells, WT but not CD44^-/-^ microglia notably increased mRNA levels of TNF-α (**Figure 4D**). Co-culture with GL261 cells slightly increased IL-1b levels in WT microglia, and surprisingly, decreased IL-1b transcription in CD44^-/-^ microglia (**Figure 4D**). A direct comparison of the levels between WT and CD44^-/-^ microglia in the presence of glioma cells revealed significantly lower levels in CD44^-/-^ microglia (**Supplementary Figure 3E**). These observations confirm a role for CD44 in the glioma cells-mediated upregulation of pro-inflammatory cytokines.

We next evaluated MMPs levels in our co-culture model. We observed a minor increase in MT1-MMP levels (1.8 fold) in CD44^+/+^ microglia cultured with GL261 cells and no change in CD44^-/-^ microglia (**Figure 4D**). However, the most striking divergence was detected for MMP9. In the presence of GL261 cells, WT microglia expressed MMP9 to a level 22-fold higher as compared to cultured alone, while no increase was detected for CD44^-/-^ microglia (**Figure 4D**). This observation was further confirmed by direct comparison between WT and CD44^-/-^ microglia in the presence of GL261 cells (**Supplementary Figure 3E**). Lack of CD44 appears to completely hinder the ability of primary microglia to upregulate MMP9 in response to tumor cells.

We further pondered on the effect these microglial secreted factors could have on glioma cells. To investigate this, we extracted and analyzed mRNA from GL261 cells co-cultured with the microglia previously described (**Supplementary Figure 4A**). TNF-α has been shown to induce IL-6 release from C6 glioma cells by modulating NFκB transcriptional activity (38). Additionally, among the most abundant secreted proteins upregulated by IL-1b is CCL2, resulting from activation of the p38 MAPK pathway (39, 40). Our results showed that both these cytokines are upregulated in GL261 cells, in the presence of microglia (**Supplementary Figure 4B**), as compared to GL261 cells cultured alone. While CCL2 levels were similar for glioma cells co-culture with WT or CD44^-/-^ microglia, IL-6 mRNA levels were reduced by half in glioma cells co-cultured with CD44^-/-^ microglia, compared to WT.

MMP9, on the other hand, apart from its primary role as ECM degrader, has been implicated in TGF-β maturation and activation (41, 42). TGF-β signaling, subsequently activates a plethora of signal transduction pathways shown to trigger, among others, MMP2 and MMP9 expression by glioma cells thereby promoting invasiveness (43–45). Our analysis however showed only a slight but not significant increase in MMP2 levels, in the presence of microglia and no difference between the WT and CD44^-/-^ conditions. No significant differences were observed for MMP9 expression (**Supplementary Figure 4B**).

### Absence of CD44 in GAMs disrupts MMP9 upregulation

**Figure 5:**
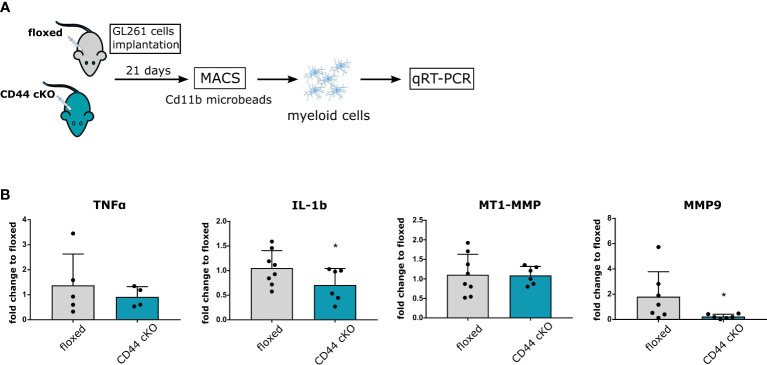
CD44 in GAMs mediates increase in MMP9. **(A)** 2 × 10^4^ GL261 cells were implanted in 5-8 weeks old floxed or CD44 cKO mice. After 21 days, myeloid cells from tumor-bearing brains were isolated by MACS with Cd11b microbeads. Total RNA was extracted and processed for qRT-PCR analysis. **(B)** mRNA levels of TNF-α, IL-1b, MMP9 and MT1-MMP. Bar charts represent mRNA levels as fold change to floxed mice. Data are presented as mean ± SD. Student’s t-Test, * p < 0.05.

To translate these findings *in vivo*, we injected intracranially GL261 cells in floxed and cKO mice. After 21 days, myeloid cells from whole brain extracts were isolated by magnetic-activated cell sorting (MACS) with Cd11b beads and qRT-PCR analysis on pro-inflammatory cytokines and MMPs were carried out (**Figure 5A**). Purity of isolated cells was verified by Iba1 staining (**Supplementary Figure 5**). In agreement with our *in vitro* data, we observed lower levels for IL-1b in myeloid cells from tumor-bearing cKO mice (**Figure 5B**). No significant effect was detected for TNF-α and MT1-MMP mRNA levels. Once more, the most striking difference was measured for MMP9, where levels in cKO mice were 25 times lower than in controls (**Figure 5B**). Altogether, our mRNA analysis *in vitro* and *in vivo* show that myeloid-cKO of CD44 severely hampers MMP9 upregulation in glioma-associated myeloid cells, and additionally affects levels of the pro-inflammatory cytokines.

## Discussion

The glioblastoma host microenvironment plays a crucial role in promoting tumor progression and invasion. Targeting the pro-tumorigenic properties of the TME therefore represents a promising option for adjuvant therapy. However, efforts in this direction still remain futile, highlighting the need for further research.

In this work, we aimed to clarify the role of the transmembrane glycoprotein CD44 in the cross-talk between malignant cells and the glioblastoma microenvironment. We show that CD44, expressed by cells of the tumor microenvironment, plays a major role in glioma invasion. Three mouse glioma lines were tested and presented disrupted invasiveness in brain slices from CD44^-/-^ mice. Invasion was affected to a different degree with the most pronounced effect observed for Sma560, followed by GL261 and finally tNSC3 cells. These differences may be explained by the difference in origin and the unique features in genetics and mutational load in these cell lines. The GL261 line was chemically induced and carries mutations in p53 and K-RAS (46). TNSC3 cells were derived from genetically engineered model, passaged *in vivo* and carry Pten and p53 mutations (47). The Sma560 model was spontaneously derived and is not yet well genetically characterized (46). Interestingly, absence of CD44 similarly affected three human cell lines as well as primary human and murine glioma cells, implying that TME-CD44-dependant invasion is a mechanism shared by various mouse and human cell models.

We further establish that spheroid invasion is particularly dependent on CD44 expressed by myeloid cells. Myeloid cells are by far the most-abundant infiltrating cells in glioma and comprise as many as 30-50% of the entire tumor mass (48). An emerging body of evidence has highlighted their pivotal role in the spread of gliomas. Their depletion reduced GBM expansion ex-vivo in cultured brain slices (49) and *in-vivo*, in a syngeneic GL261 model (37). A direct promoting effect of microglia on glioma invasion has been shown in the Boyden chamber assay (50). Here we demonstrate that this effect is dependent on microglial CD44. CD44^-/-^ microglia failed to increase the number of invading GL261 cells compared to no-cells control, contrary to WT microglia. Hence, CD44-mediated invasion appears to be one crucial aspect of the importance of myeloid cells in GBM. This is in agreement with a recent study suggesting that the correlation between poor prognosis and high CD44 levels in glioblastoma is linked to the presence of CD44-expressing GAMs and to their pro-tumorigenic transformation (18).

Since our results showed no evident link between CD44 and myeloid cells recruitment or morphology transformation, a role in the regulation of protumorigenic gene expression appeared more likely. One report by Qadri and colleagues has highlighted a role of CD44 as a regulator of TLR2 signaling in macrophages in the context of osteoarthritis. Stimulation of TLR2 increased the production of pro-inflammatory cytokines while antibodies against CD44 or its silencing by siRNA led to a significant reduction in those levels following TLR2 activation (34). This consolidated previous works showing an implication of CD44 in TNF-α and IL-1b production and release in monocytes (32, 33). Furthermore, upon TLR2 stimulation with its natural agonist zymozan, CD44 and TLR2 co-immunoprecipitated, highlighting direct association of the two receptors under these conditions (35). Using a direct TLR2 agonist, Pam3SCK4, we were able to show a strong deleterious effect of CD44 knock-out on microglial TNF-α and IL-1b mRNA levels *in vitro*. Furthermore, we observed similar results for microglia co-cultured with GL261 cells and for glioma-associated myeloid cells from cancer-bearing mice. These results further establish a role for CD44 on myeloid cells in glioma-associated TLR2 signaling activation.

Pro-tumorigenic functions of GAMs additionally involve production of matrix metalloproteinases MMP9 and MT1-MMP, which promote GBM invasion (36, 51–53). Here we demonstrate a strong implication of CD44 in the MMP9 upregulation in GAMs. In our assay we observed a drastic increase in MMP9 levels upon co-culture with GL261 cells, consistent with previous studies (36), which was completely abolished in CD44^-/-^ microglia. Similarly, GAMs isolated from cKO mice presented with significantly lower MMP9 levels compared to those isolated from controls. It is interesting to note that, upon direct TLR2 stimulation, CD44^-/-^ microglia were still able to increase MMP9 production, while this ability was completely abolished in the presence of glioma cells. This suggest that in the cross-talk between microglia and tumor cells, there is a MMP9-promoting pathway complementary to TLR2. In this context, a recent report demonstrates that IL-6 induces MMP9 production in macrophages by activating the STAT and MAPK signaling pathways (54). When incubated with exogenous IL-6, macrophages responded with a dose-dependent increase in MMP-9 mRNA levels. In our co-culture experiments, GL261 cells produced significantly less IL-6 in the presence of CD44^-/-^ microglia, which we attributed to lower myeloid-derived pro-inflammatory cytokines. This reduction in glioma-derived IL-6, together with altered TLR2 signaling, could concomitantly hinder microglial MMP9 production. This would imply that in the context of glioma, myeloid CD44 regulates both directly and indirectly MMP9 increase, which could explain the observed drastic effect on this proteinase.

We did not observe any major differences in MT1-MMP levels in cKO mice compared to controls. One explanation for this could be provided by two studies from the Kettemann group showing that MMP9 induction requires activity of the TLR2/TLR6 heterodimer, while MT1-MMP production is mediated equally by the TLR2/TLR6 and TLR1/TLR2 complexes (36, 51). It is tempting to speculate that myeloid CD44 is predominantly implicated in the TLR2/TLR6 pathway.

Altogether, failure to upregulate MMP9 and reduction in pro-inflammatory cytokines indicate an altered pro-tumorigenic polarization of CD44^-/-^ myeloid cells in response to GBM. In agreement with this, a recent study has found that HA synthetized by glioma cells binds CD44 on myeloid cells thereby triggering their immunosuppressive polarization (55). Authors showed that by blocking CD44 on macrophages, the latter acquire an M1 phenotype resulting in reduced glioma proliferation and migration and increased survival of tumor-bearing mice.

Even though absence of CD44 strongly disrupted glioma invasion in brain slices and *in vitro*, cell invasion from DKO11804 and GL261-derived tumors was only mildly affected in myeloid CD44-deficient syngeneic hosts, despite the significant differences of both cell lines in respect to tumor growth and infiltration of the surrounding tissue. This once again demonstrates the complexity of *in vivo* cell-cell interactions and limitations of *in vitro* models. While our results demonstrate a disrupted pro-tumorigenic profile of CD44^-/-^ microglia in response to glioma cells, it appears that *in vivo*, these alterations are not sufficient to significantly affect glioma invasion or increase survival. These observations suggest the occurrence of compensatory mechanisms *in vivo*, although the implicated cell types and pathways remain to be explored. The simplest explanation would be that the even though drastically reduced, MMP9 levels in CD44^-/-^ GAMs are still sufficient to promote tumor invasion. It is possible to imagine that myeloid-derived MMP9 is accumulating in the TME and reaches a certain threshold necessary to promote glioma infiltration. This would be in agreement with our MRI analysis showing that tumor volume is initially lower in cKO compared to floxed mice, but with cancer progression this advantage diminishes and ultimately is not sufficient to confer survival benefit. One could also imagine, that MMP9 released by other cell types in the tumor bulk like endothelial cells, neutrophils and glioma cells compensates, at least in part for its reduction in GAMs. Finally, other factors could potentially functionally compensate for MMP9 reduction. As a biomarker for numerous cancers, MMP9 has versatile roles (56). Its active form degrades various matrix proteins modulating the extracellular matrix and it is equally involved in the cleavage and processing of a plethora of bioactive molecules. As with other types of cancer, mechanisms implicated in gliomagenesis and glioma progression are regulated by multiple parallel pathways, thus other metalloproteinases, cell receptors or signaling molecules could very well act concurrently with MMP9 in promoting GBM invasiveness. This once more highlights the necessity of combined therapeutic strategies targeting simultaneously malignant cells and cells from the microenvironment.

In summary, our study demonstrates that CD44 is an important factor in the cross-talk between tumor and host cells. Our results highlight a role of CD44 expressed specifically by myeloid cells from the TME, in glioma invasion. CD44 knock-out in myeloid cells affected their ability to upregulate TNF-α and IL-1b, and most notably, to increase MMP9 production in response to gliomas. This in turn affected the migration ability of glioma cells in brain slices and *in vitro*. Targeting CD44 in myeloid cells could modulate their polarization in response to glioma and impede their distinctive pro-tumorigenic ability. CD44 thus holds a therapeutic potential as stroma-directed therapy target for glioblastoma.

## Materials and methods

### Animals

All animal experiments were approved by the responsible authority for animal experiments (Regierungspräsidium Karlsruhe, Germany) and performed in conformity with the German Law for Animal Protection (animal license number: G-122/16, G-171/21).

CD44^-/-^ (full name B6-Cd44tm1Mak) mice were kindly provided by Dr. Tak W. Mak (Department of Immunology, University of Toronto, Canada) and already described (24). C57Bl/6N wild-type mice were purchased from Janvier Labs. Ve-Cadherin CreER^T2^ mice were kindly provided by Prof. Ralf Adams (Max Planck Institute for Molecular Biomedicine, Department of Tissue Morphogenesis, University of Münster), Csf1R-Cre (29) and GFAP-Cre (27) were provided by Prof. Michael Platten (Department of Neurology, University Heidelberg).

CD44^fl/fl^ floxed mice were crossed with Csf1R-Cre to obtain Csf1R-Cre/CD44^fl/fl^ mice and with GFAP-Cre mice to obtain GFAP-Cre/CD44^fl/fl^ mice. Ve-Cadherin CreER^T2^ were crossed with CD44^fl/fl^ floxed mice to obtain Ve-Cadherin CreER^T2^/CD44^fl/fl^ mice. To induce recombination of floxed alleles, 4-week-old mice were treated with 1 mg/20g body weight Tamoxifen in peanut oil for five consecutive days. As a control, mice were treated with peanut oil.

### Cells

GL261 and SMA560 cells were provided by Prof. Michael Platten (Department of Neurology, University Heidelberg). LN319 were provided by Prof. Wolfgang Wick (University Hospital Heidelberg). U87MG and NMA59 were provided by Prof. Michael Weller (Department of Neurology, University Zurich) and Prof. Ana Martin-Villalba (DKFZ, Heidelberg), respectively. tNSC3 cells were generated in our laboratory and described in (47). Human and murine primary glioblastoma cultures were generated in our laboratory (23, 47, 57).

Murine and human glioma cell lines were cultivated in DMEM supplemented with 10% FBS, 2 mM L-glutamine and 100 U/ml penicillin/streptomycin at 37°C and 5% CO_2_. Primary murine glioma cells (DKO11804) were cultured as spheroids in DMEM/F12 medium (Life Technologies) supplemented with N2, 20 ng/ml of each EGF and FGFb (Promokine), 2 mM L-glutamine and 100 U/ml penicillin/streptomycin, at 37°C and 5% CO_2_. Primary human cells were cultured in serum-free neurobasal medium, containing N 2 supplement (Life Technologies, 17502048), 20 ng/ml of both EGF and FGFb, 2 mM L-glutamine and 1% penicillin/streptomycin (100 U/ml) at 37°C and 5% CO_2_.

### Ex vivo brain slices assay

Studies of invasiveness on brain slices were performed as previously described (23). Briefly, 6–8 week old C57Bl/6 wild-type, CD44^-/-^, or cKO mice were euthanized, the brain was isolated and the cerebellum removed. 350 μm thick coronal slices were cut with a vibratome (Leica VT1200 S) with a speed of 0.2 mm/s and collected on 0.4-µm pore size filters (Millipore, PICM03050) in 6-well plates, in brain slice medium (MEM (Sigma, M2279), 25% heat-inactivated horse serum (Life Technologies, 26050070), 25 mM HEPES (Sigma, H0887-100 mL), 1 mM L- glutamine (Sigma, G7513), 5 mg/ml glucose (Sigma, G8769), 100 U/ml penicillin/streptomycin (Sigma, P4333)). Slices were cultured at 37 °C and 5% CO_2._ Medium was exchanges 18-24 h after culture initiation and then was refreshed every other day.

Murine and human glioma cell lines cultivated in serum-containing medium were trypsinized and counted. 1 × 10^6^ cells/ml PBS were incubated with 5 μl lipophilic dye DiD (DiIC18 (5); 1,1′-dioctadecyl-3,3,3′,3′-tetramethylindodicarbocyanine, 4-chlorobenzenesulfonate salt) (5 µg/mL; Biotium, 60014) for 30 min at 37 °C. Cells were washed twice in PBS and then 500 cells/well were seeded in a flat-bottom 96-well plate coated with 50 μl low melt agarose (Genaxxon, M3049.0010; 1% in PBS). Human and murine primary cells were cultivated as spheroids in serum-free medium as described previously (25). For spheroids formation cells were labeled as described above and seeded in agarose-free U-bottom 96-well plates (500 cells/well; Greiner, 650185).

After 48 hours of culture 8–10 spheroids per brain slice were manually implanted using a blunt Hamilton syringe (701 N; 10 μl; 26 s/51/3) and a binocular microscope. After 48 hours slices were fixed in 4% PFA and processed for tissue clearing according to the SeeDB protocol (58). Imaging was performed with a Nikon Eclipse Ti (Nikon, Düsseldorf) and Z-stack images were transformed to a maximum projection image by using ImageJ (59).

### Primary microglia isolation

Mixed glial cultures were obtained from newborn P0-P4 pups. Brains were isolated and meninges removed. Cortices were mechanically dissociated using a glass pestle and passed through a 100 μm cell strainer (Falcon, 352360). All cortices were pooled together, washed with plain DMEM medium, and seeded on 6P or 24P plates (4×6P wells or 6×24P wells per pup in pool). Debris were washed off the next day with PBS and medium refreshed. Mixed cultures were then cultivated in DMEM supplemented with 10% FBS, 2 mM L-glutamine, 100 U/ml penicillin/streptomycin, at 37 °C and 5% CO_2._ After 3 weeks, microglia were isolated by mild trypsinization (60).

### Primary astrocytes isolation

Primary mouse astrocytes were obtained from newborn P0-P4 pups. After removal of the meninges under a binocular microscope, the cortices of both hemispheres were mechanically minced and passed through a 100 μm cell strainer (Falcon, 352360). Cells were seeded into a culture-treated T75cm2 flask coated FBS, 2 mM L-glutamine, 100 U/ml penicillin/streptomycin. One day after isolation, the medium was exchanged. A confluent monolayer was obtained after one week culturing in a standard incubator at 37°C and 8% CO2. Astrocytes were split at a confluence of 90-95%.

### Primary mouse brain endothelial cells isolation

Primary mouse brain endothelial cells (mBECs) were isolated from four- months old mice. Brains were dissected in ice-cold dissociation buffer (153 mM NaCl, 5.6 mM KCl, 1.7 mM CaCl2, 1,2mM MgCl2, 15 mM HEPES, 1% BSA). Cerebellum and meninges were removed and the brain tissue was homogenized in using a tight homogenizer (ratio brain: dissociation buffer 1:2). The homogenate was transferred to a 15 mL falcon tube and centrifuged at 300g for 10 min at 4°C. The supernatant was removed. Dissociation buffer and 0.75% collagenase Type 2 were added at a ratio of 1:1, mixed incubated at 37°C for 1 hour while shaking. The mixture was centrifuged at 400g for 10 min at 4°C. Pellet was resuspended in 12 mL 25% BSA/PBS and centrifuged at 1700g for 30 min at 4°C. The supernatant and the white layer of myelin on top were removed and the pellet was gently resuspended in 4 mL dissociation buffer and transferred to a fresh 15 mL falcon tube. Collagenase/Dispase (1 mg/mL) and Dnase 1 (1 µg/mL) were added and incubated for 15 min at 37°C while shaking. After a last centrifugation step at 300g for 5 min at RT, the pellet was resuspended in 2 mL complete mBECs medium (MCDB 131 medium (Thermo Fischer, 10372019) supplemented with 20% FCS, 2 mM L-glutamine, 100 U/mL penicillin, 100 µg/mL streptomycin, 5 mM heparin, 50 µg/mL endothelial cell growth factor, 1 g/L sodium bicarbonate, 4 µg/mL puromycin (Gibco, A11138-03)) and plated on rat tail collagen I (1 mg/mL Corning, 354236) coated chamber slides. After having attached, cells were washed with PBS and incubated with mBECs medium. Cells were maintained in culture for one week and then subjected to immunofluorescence staining.

### Bone marrow derived macrophages isolation

Bone marrow-derived macrophages were isolated by flushing femur and tibia of adult mice as previously described (61). Briefly, after erythrocyte lysis and a washing step with plain DMEM, cells obtained from one leg were seeded on uncoated plates and cultured in RMPI 1640 GlutaMAX (Thermo Fisher Scientific) supplemented with 10% FBS, 100 U/ml penicillin/streptomycin, 50 mM β-mercaptoethanol and 30% L929-MCSF-conditioned medium.

### Boyden experiments

0.8-µm pore size filters (Corning FluoroBlok, 351152) were coated with Matrigel (Corning, 356231) and transferred to 24-well plates containing primary microglia 48 hours post mild trypsinization. 5 × 10^4^ GL261 were seeded on top of the filters. Cells were cultured together for 24 hours. Inserts were then washed with PBS and fixed in 4% PFA for 20 min at RT. After washing with PBS, migrating cells were stained with DAPI and Phalloidin (Abcam, ab235138). Imaging was performed with a confocal microscope (Zeiss LSM 700) with a 10x objective and analysis was done with ImageJ.

### Co-culture experiments

2 × 10^5^ GL261 were seeded on 0.4-µm pore size filters (Falcon, 353493) in DMEM supplemented with 10% FBS, 2 mM L-glutamine, 100 U/ml penicillin/streptomycin. After 24 hours inserts were transferred in 6-well plates containing primary microglia 48 hours post mild trypsinization. Cells were cultured together for additional 24 hours. Microglia were then washed with PBS, collected in QIAzol Lysis Reagent and processed with the RNeasy kit (Quiagen, 73404) according to the manufacturer’s protocol.

### PAM3CSK4 stimulation

Primary microglia in 6-well plates were stimulated with PAM3CSK 4 (10ng/mL Cayman Chemical, 24126) for 6 hours. Cells were then washed with PBS, collected in QIAzol Lysis Reagent and processed with the RNeasy kit (Quiagen, 73404).

### Intracranial injections

Mice were anesthetized with isoflurane. Orthotopic injections were performed with a motorized stereotaxic instrument (Neurostar). 1 × 10^6^ DKO11804 or 2 × 10^4^ GL261 cells were injected in 2 μl PBS 2 mm lateral (right hemisphere) and 3 mm ventral to the bregma with a speed of 0.2 μl/min, using a 10-µL precision microsyringe (World Precision Instruments, Inc, Sarasota, FL, USA) with a 34G needle. Five to eight weeks old C57Bl/6 wild-type, CD44^-/-^, CD44^fl/fl^ or Csf1r-Cre; CD44^fl/fl^ mice were used as recipients. Tumor volume was monitored with MRI. Mice injected with DKO11804 were sacrificed upon displaying termination criteria such as loss of >20% body weight, neurological deficits or poor general condition. Survival was assessed by means of Kaplan–Meier estimate. Mice injected with GL261 cells were sacrificed after 14 days for immunohistochemical analysis and after 21 days for MACS sorting of GAMs. For immunofluorescence and immunohistochemistry analysis mice were euthanized with carbon dioxide, perfused with PBS, and the brains were fixed overnight at 4°C in 4% PFA.

### MRI

MRI was carried out by our small animal imaging core facility in DKFZ using a Bruker BioSpec 3Tesla (Ettlingen, Germany) with ParaVision software 360 V1.1. For the imaging, mice were anesthetized with 3.5% sevoflurane in air. For lesion detection T2 weighted imaging were performed using a T2_TurboRARE sequence: TE = 48 ms, TR = 3350 ms, FOV 20x20 mm, slice thickness 1 mm, averages = 3, Scan Time 3m21s, echo spacing 12 ms, rare factor 8, slices 20, image size 192x192. Tumor volume was measured using a T1-FLASH sequence: TE = 3ms, TR = 500ms, FOV 20x20 mm, slice thickness 1 mm, slices 20, Flip angle 70, averages = 3, resolution = 0,104 mm. Tumor volume was determined by manual segmentation using Bruker ParaVision software 6.0.1

### MACS

The protocol for isolation of myeloid cells from tumor-bearing mice was adapted from (62). Mice were euthanized with CO_2_ and perfused with cold PBS. Brains were dissected, cerebellum and olfactory bulbs were removed. Brains were then mechanically dissociated and transferred into dissociation buffer (Lebovitz (Gibco, 21083027), cell dissociation solution (Millipore, S040C), 0.5mM EDTA, 50 U/mL DNAse, 1.2 U/mL Dispase II) and incubated on a rotating block at 37° for 1h. Samples were then further mechanically dissociated with a P1000 and then a Pasteur pipette, filtered through 70 µm filter and centrifuged at 1500 rpm for 10 min. Erythrocytes were lysed with ACK solution for 3 min and cells were centrifuged for another 10 min at 1500 rpm. Myelin was removed by 45 min centrifugation in 35% Percol solution. MACS isolation of myeloid cells was performed with CD11b microbeads (Milteny Biotec, 130-049-601) according to the manufacturer’s protocol. Isolated cells were then washed with PBS, collected in QIAzol Lysis Reagent and processed with the RNeasy kit (Quiagen, 73404).

### Immunohistochemistry and immunofluorescence

PFA fixed cells or 6-µm formalin fixed paraffin-embedded sections were stained according to standard immunohistochemistry protocols. The following antibodies were used: mCherry (abcam, ab167453), Iba1 (Wako, 01919741), Cd44 (Abcam 119863), CD31 (Abcam, ab28364), GFAP (BioLegend, 644701). Secondary antibodies were from Thermo Fischer Scientific. For brain tissue analysis, images were acquired with Zeiss Axio-Scan.Z1 using ZEN software (Zeiss, Oberkochen, Germany). Cultured cells were imaged with a MEA53100 Eclipse Ti-E inverted microscope (Nikon, Japan). Analysis were performed using ImageJ.

### Quantitative real-time PCR

For quantitative gene expression analysis, 40 cycles of real-time PCR was performed on the StepOnePlus real-time detection system (Applied Biosystems). Every PCR reaction was carried out in duplicates with 2.5 ng of cDNA in a final volume of 12.5 μL Power SYBR Green PCR Master Mix (Applied Biosystem). StepOne Software v2.2 was used for data analysis. GAPDH protein were used as housekeeping gene to normalize target gene expression. Primer sequences are given in **Supplementary Table 1**. mRNA expression was calculated using the ΔΔCt method. Data are presented as fold target gene expression change relative to control.

### Statistical analysis

All experiments were performed in at least three independent replicates and analysis were performed blinded to genotype or condition when possible. All statistics were calculated with GraphPad Prism v.7 (GraphPad Software Inc.). Data are presented as mean ± SD. Parametric testing was done with the Student’s t-Test. Comparisons between multiple groups were done using One-way ANOVA with the Tukey’s multiple comparisons test. *p < 0.05; **p < 0.01; ***p < 0.001; ****p < 0.0001; “n.s.” implies a non-significant P value.

## Data availability statement

The original contributions presented in the study are included in the article/**Supplementary Material**. Further inquiries can be directed to the corresponding author.

## Ethics Statement

The animal study was reviewed and approved by Regierungspräsidium Karlsruhe, 76247 Karlsruhe, Germany.

## Author contributions

EI wrote the manuscript original draft. EI, BC, HP and PA designed the study. EI, BC, TE, conducted the experiments and data analysis. SL contributed to the experiments and technical support. PB and JM contributed to the experiments, interpretation and data analysis. VE and MJ conducted MRI experiments, interpretation and data analysis. VO-R contributed to the study concept, writing and editing of the manuscript. PA supervised the project and provided the funding. All authors reviewed the manuscript. All authors contributed to the article and approved the submitted version.

## Acknowledgments

We thank Bettina Kast for technical assistance, Ralf H Adams, Tak W. Mak and Michael Platten for providing genetically modified mouse strains, the laboratory of Stefan Liebner for advice in preparing mBECs, Barbara Böck for administrative handling of animal licenses and Susanne Kleber and Ana Martín-Villalba for access to stereotactic devices. Support by the DKFZ Light Microscopy Facility is gratefully acknowledged. The authors also thank the DKFZ Central Animal Laboratory for animal care and the DKFZ Flow Cytometry Facility for cell sorting.

## Conflict of interest

The authors declare that the research was conducted in the absence of any commercial or financial relationships that could be construed as a potential conflict of interest.

## Publisher’s note

All claims expressed in this article are solely those of the authors and do not necessarily represent those of their affiliated organizations, or those of the publisher, the editors and the reviewers. Any product that may be evaluated in this article, or claim that may be made by its manufacturer, is not guaranteed or endorsed by the publisher.
