# Supplementary material for: CD44 expressed by myeloid cells promotes glioma invasion
Source: Front Oncol. 2022 Aug 4;12:969787. doi: 10.3389/fonc.2022.969787 (PMC9386454; doi:10.3389/fonc.2022.969787)
Supplement: Supplementary file 1 [file DataSheet_1.pdf]

# *Supplementary Material*

| Transcript | forward sequence 5‘-3‘ | reverse sequence 5‘-3 | Reference |
|---|---|---|---|
| GAPDH | TGAAGCAGGCATCTGAGGG | CGAAGGTGGAAGAGTGGGAG | |
| TNFα | AGGCACTCCCCCAAAAGATG | GCTCCTCCACTTGGTGGTTT | |
| IL-1b | TGCCACCTTTTGACAGTGATG | AAGGTCCACGGGAAAGACAC | |
| CD44 | GCACTGTGACTCATGGATCC | TTCTGGAATCTTGAGGTCTCC | (1) |
| TLR2 | CCCTGTGCCACCATTTCC | CCACGCCCACATCATTCTC | (2) |
| MMP9 | AAGGGGCGTGTCTGGAGATT | GGTACTGGAAGATGTCGTGTGAGT | (3) |
| MT1-MMP | GTGCCCTATGCCTACATCCG | CAGCCACCAAGAAGATGTCA | (2) |
| MMP2 | CTGGAATGCCATCCCTGATAA | CAAACTTCACGCTCTTGAGACTTT | (3) |
| CCL2 | TTTTGTCACCAAGCTCAAGAGA | ATTAAGGCATCACAGTCCGAGT | (4) |
| IL-6 | TGAACAACGATGATGCACTTG | CTGAAGGACTCTGGCTTTGTC | |

**Supplementary Table 1**. List of Primers used for qualitative Real-Time PCR.

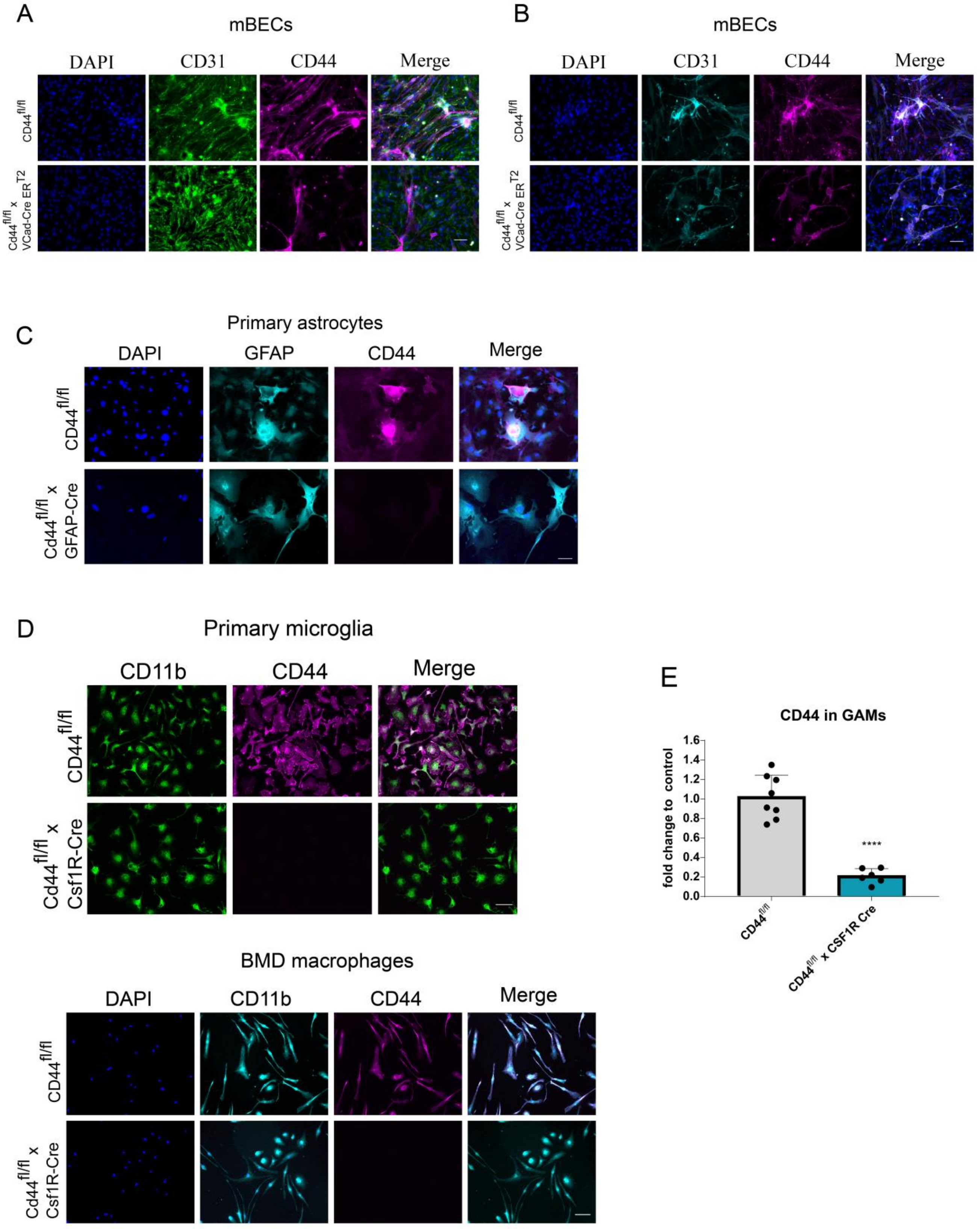
A
mBECs
DAPI
CD31
CD44
Merge
CD44fl/fl
Cd44fl/fl x VCad-Cre ERT2
B
mBECs
DAPI
CD31
CD44
Merge
CD44fl/fl
Cd44fl/fl x VCad-Cre ERT2
C
Primary astrocytes
DAPI
GFAP
CD44
Merge
CD44fl/fl
Cd44fl/fl x GFAP-Cre
D
Primary microglia
CD11b
CD44
Merge
CD44fl/fl
Cd44fl/fl x Csf1R-Cre
BMD macrophages
DAPI
CD11b
CD44
Merge
CD44fl/fl
Cd44fl/fl x Csf1R-Cre
E
CD44 in GAMs
fold change to control
1.6
1.4
1.2
1.0
0.8
0.6
0.4
0.2
0.0
****
CD44fl/fl
CD44fl/fl x CSF1R Cre

**Supplementary Figure 1.** Conditional mouse lines validation.

(**A-B**) Validation of endothelial-specific CD44 depletion in $CD44^{fl/fl}$×Ve-Cadherin $CreER^{T2}$ mice. Primary mouse brain endothelial cells (mBEC) were isolated from floxed controls and $CD44^{fl/fl}$×Ve-Cadherin $CreER^{T2}$ mice and stained for CD44 (magenta), CD31(green), GFAP (cyan) and counterstained with DAPI (blue). Scale bars 50µm.(**A**) Representative images from $CD44^{fl/fl}$ and $CD44^{fl/fl}$×Ve-Cadherin $CreER^{T2}$ mice. In mBECs from floxed mice, the majority of cells express CD44. In the $CD44^{fl/fl}$×Ve-Cadherin $CreER^{T2}$ model, the number of CD44-positive cells is strongly reduced. (**B**) Representative images from $CD44^{fl/fl}$ and $CD44^{fl/fl}$×Ve-Cadherin $CreER^{T2}$ mice showing the presence of CD44-positive contaminating astrocytes. In $CD44^{fl/fl}$ cultures, CD44 staining does not exclusively colocalize with GFAP staining. Conversely, in $CD44^{fl/fl}$×Ve-Cadherin $CreER^{T2}$, all CD44-positive cells are also positive for GFAP indicating those are astrocytes which tightly associate with endothelial cells. (**C**) Validation of astrocyte-specific CD44 depletion in $CD44^{fl/fl}$×GFAP-Cre mice. Mouse primary astrocytes were isolated from floxed controls and $CD44^{fl/fl}$×GFAP-Cre newborn mice, stained for GFAP (cyan) and CD44 (magenta) and counterstained with DAPI (blue). No CD44 staining was observed in $CD44^{fl/fl}$× GFAP-Cre cultures. Double-positive astrocytes were present only in $CD44^{fl/fl}$ controls. Scale bar 50µm. (**D**) Validation of myeloid-specific CD44 depletion in $CD44^{fl/fl}$×Csf1R-Cre mice. Upper panel: Mouse primary microglia were isolated from floxed controls and $CD44^{fl/fl}$×Csf1R-Cre newborn mice and stained for CD11b (green) and CD44 (magenta). Double-positive microglia were present in controls but not in $CD44^{fl/fl}$×Csf1R-Cre cultures. Lower panel: Bone-marrow derived (BDM) macrophages were isolated from floxed controls and $CD44^{fl/fl}$×Csf1R-Cre newborn mice, stained for CD11b (cyan), CD44 (magenta) and counterstained with DAPI (blue). Double-positive macrophages were present in controls but not in $CD44^{fl/fl}$×Csf1R-Cre cultures. Scale bars 50µm. (**E**) CD44 levels in GAMs isolated by MACS from tumor bearing floxed and $CD44^{fl/fl}$×Csf1R-Cre mice were evaluated by qRT-PCR. CD44 mRNA levels in $CD44^{fl/fl}$×Csf1R-Cre mice were 5-fold lower than in controls. Data are presented as mean ± SD. Student's t-Test, ****$p < 0.0001$.

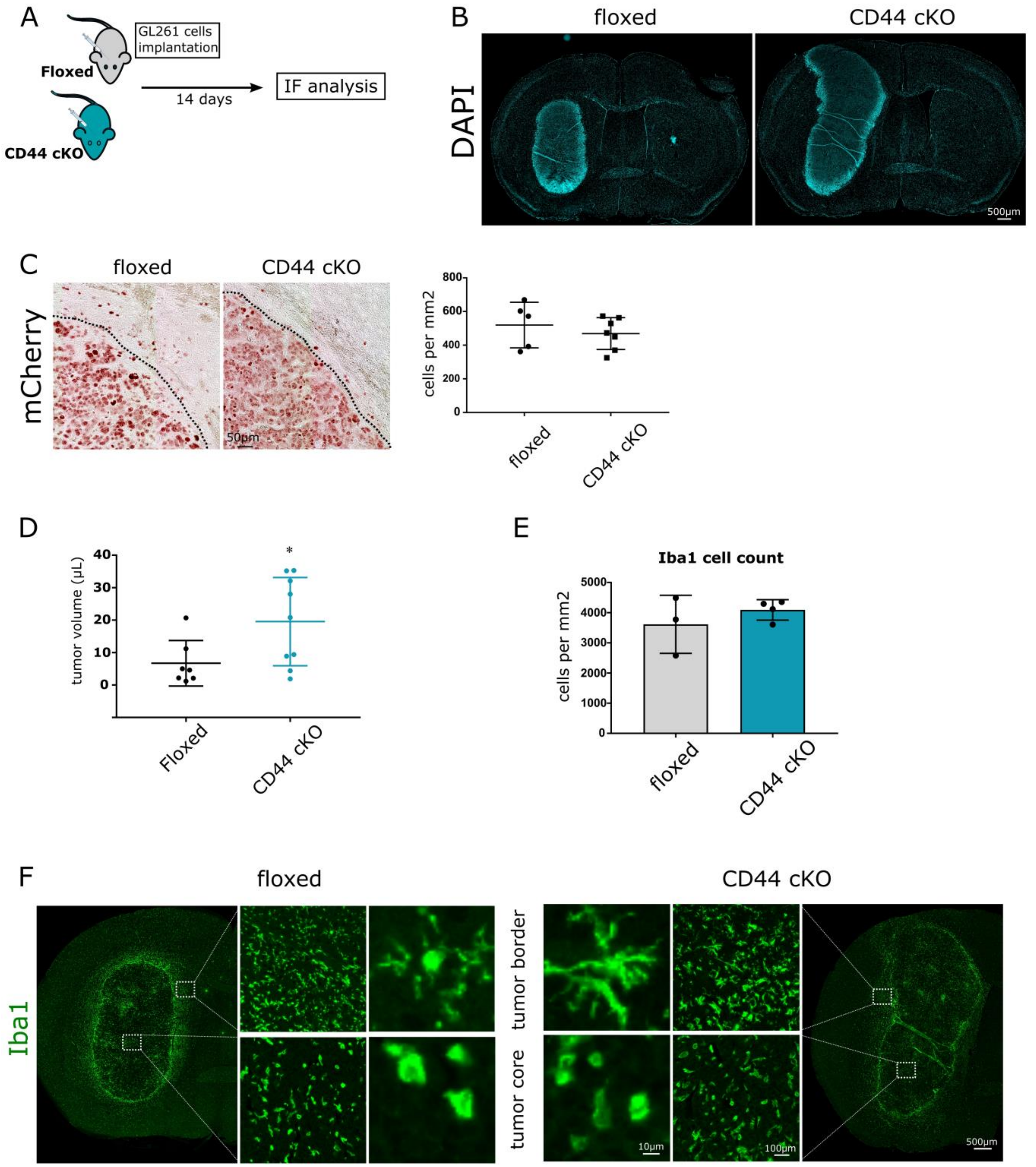


**Supplementary Figure 2.** Effects of Myeloid-specific CD44 knock-out on GL261 tumors in-vivo.

(**A**) $2 \times 10^4$ GL261 mCherry labelled cells were implanted in 5-8 weeks old floxed or cKO mice. Analysis were performed after 14 days. (**B**) Representative images of brains from floxed and cKO tumor-bearing mice stained with DAPI showing a bulk growth pattern. (**C**) Left-Representative images of tumors from floxed and cKO mice stained with mCherry. Dashed lines delimit the tumor

core. Right-Quantification of the number of mCherry-positive tumor cells migrating beyond the tumor border. The tumor border was manually drawn and the number of cancer mCherry-positive cells spreading in a radius of 300 microns was counted. (**D**) Tumor volume at 14 days, measured by MRI. Graph represent tumor volume (µl) for individual mice calculate from T2-weighted images. Data are presented as mean±SD. Student's t-Test, * $p < 0.05$. (**E-F**) Myeloid cells recruitment and morphology at endpoint. (**E**) Graphs depict the number of Iba1 positive cells per $mm^2$ in the tumor core. (**F**) Representative images of Iba1- positive cells. At the tumor border, labelled cells present with ramified morphology, whereas within the tumor core their morphology is amoeboid.

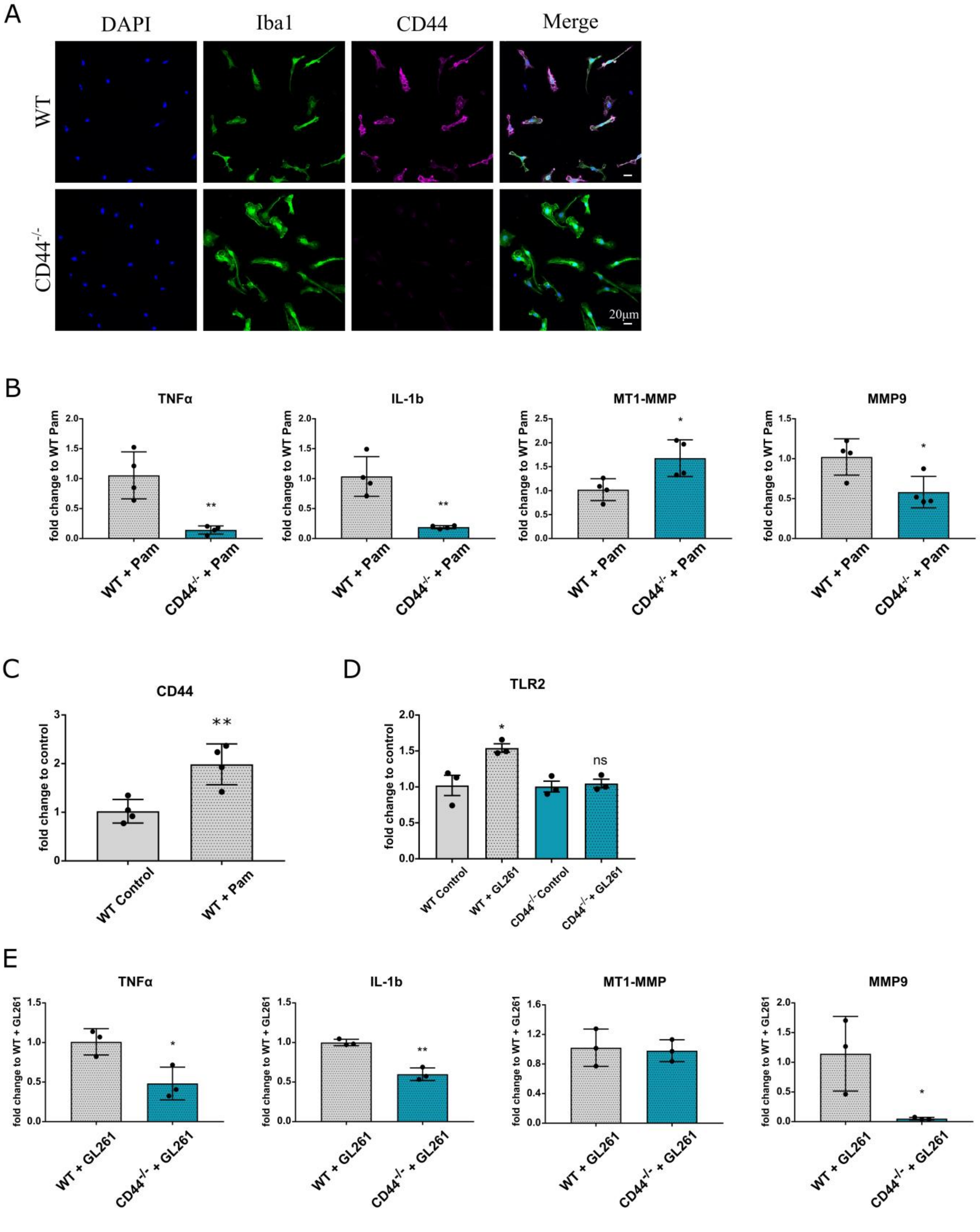


**Supplementary Figure 3.** CD44 and TLR2 signaling.

(**A**) Validation of the purity and CD44 expression in isolated primary microglia from WT and $CD44^{-/-}$ mice. Primary microglia were stained for Iba1 and CD44 and counterstained with DAPI. The representative image shows that all cells in the culture (DAPI) are positive for Iba1(green). Only cells isolated from WT mice co-express CD44 (magenta). (**B**) mRNA levels of TNF-α, IL-1b, MT1-MMP and MMP9 in primary WT or $CD44^{-/-}$ microglia after 6 hours of stimulation with the TLR2 agonist Pam3CSK4. Graph represents mRNA levels as fold change WT + Pam condition. Data are presented as mean±SD. Student's t-Test, * $p < 0.05$, ** $p < 0.01$. (**C**) mRNA levels of CD44 in primary microglia after 6 hours of stimulation with the TLR2 agonist Pam3CSK4. Graph represents mRNA levels as fold change to unstimulated control. Data are presented as mean±SD. Student's t-Test, ** $p < 0.01$. (**D**) mRNA levels of TLR2 in primary WT or $CD44^{-/-}$ microglia, after 24 hours co-culture with GL261 cells or "no cells" control condition. Graph represents mRNA levels as fold change to corresponding control. Data are presented as mean±SD. Student's t-Test, * $p < 0.05$ vs control, ns = not significant. (**E**) mRNA levels of TNF-α, IL-1b, MT1-MMP and MMP9 in primary WT or $CD44^{-/-}$ microglia after 24 hours co-culture with GL261 cells. Graph represents mRNA levels as fold change WT + GL261 condition. Data are presented as mean±SD. Student's t-Test, * $p < 0.05$, ** $p < 0.01$.

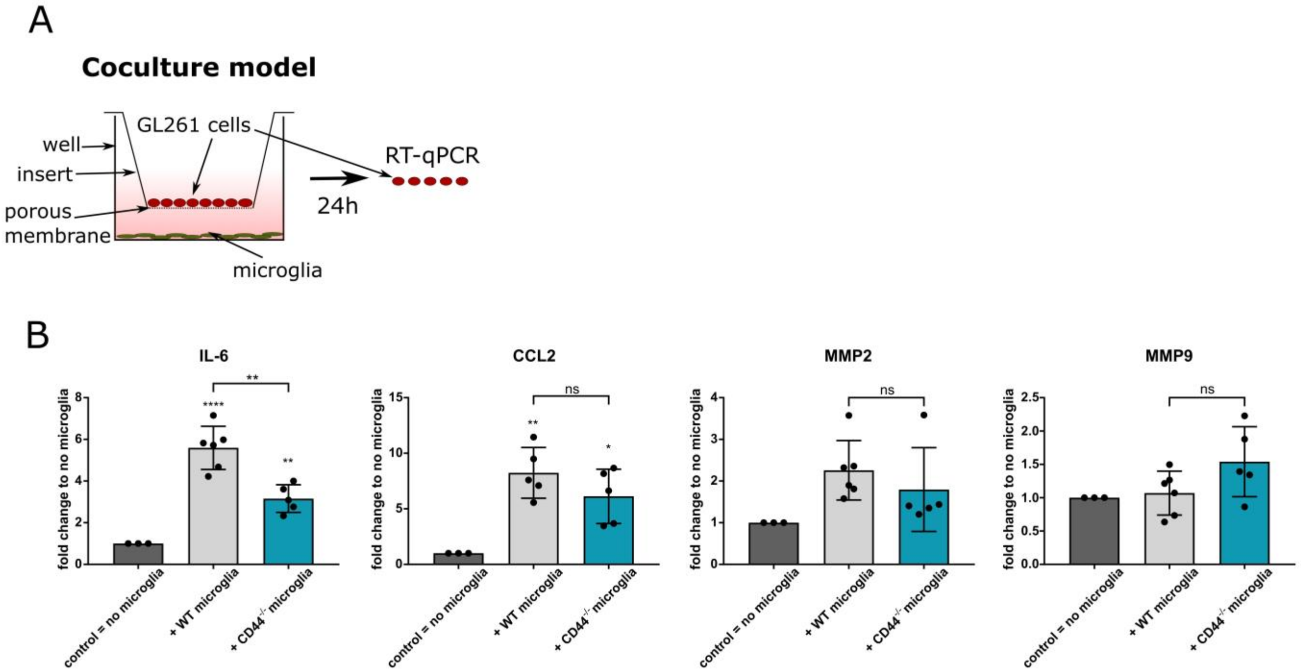


**Supplementary Figure 4.** qRT-PCR on GL261 cells co-cultured with WT or CD44$^{-/-}$ microglia.

(**A**) Schematic representation of the co-culture experiment. GL261 cells were co-cultured for 24 hours with primary microglia isolated from WT or CD44$^{-/-}$ mice. Total RNA from GL261 cells was extracted and processed for qRT-PCR analysis. GL261 cells cultured without microglia in the bottom compartment were used as control. (**B**) mRNA levels of IL-6, CCL2, MMP2 and MMP9 as fold change to culture without microglia. Data are presented as mean±SD. Student's t-Test, $**p < 0.01$, $****p < 0.001$, ns = not significant.

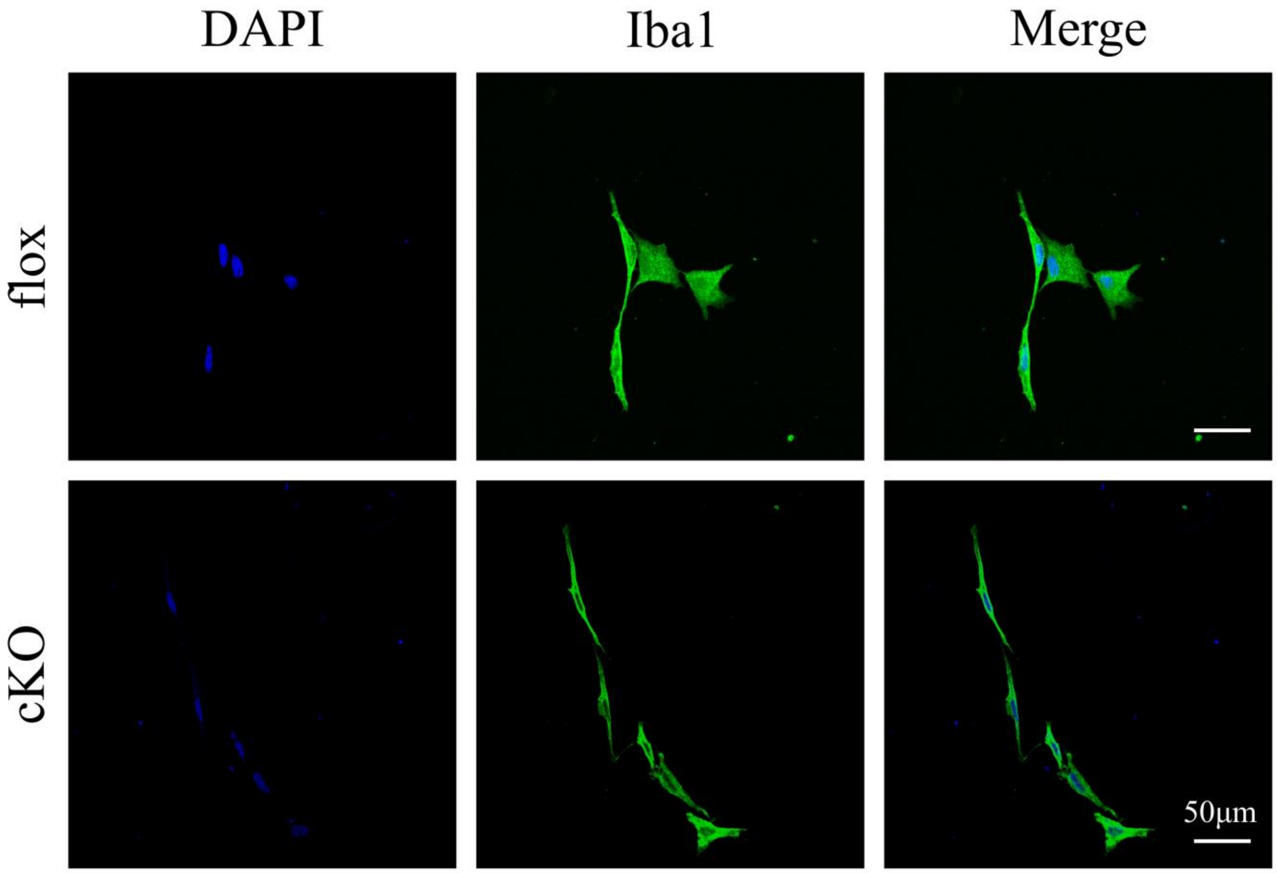


**Supplementary Figure 5.** Evaluation of the purity of cells isolated by MACS. 2 × $10^4$ GL261 cells were implanted in 5-8 weeks old floxed or CD44 cKO mice and myeloid cells from tumor-bearing brains were isolated by MACS with Cd11b microbeads. Isolated cells were stained for Iba1 (green) and counterstained with DAPI (blue). The representative image shows that all purified cells (DAPI) are positive for Iba1.

## References


1. Shatirishvili M, Burk AS, Franz CM, Pace G, Kastilan T, Breuhahn K, Hinterseer E, Dierich A, Bakiri L, Wagner EF, et al. Epidermal-specific deletion of CD44 reveals a function in keratinocytes in response to mechanical stress. *Cell Death Dis* (2016) 7: doi: 10.1038/CDDIS.2016.342

2. Vinnakota K, Hu F, Ku MC, Georgieva PB, Szulzewsky F, Pohlmann A, Waiczies S, Waiczies H, Niendorf T, Lehnardt S, et al. Toll-like receptor 2 mediates microglia/brain macrophage MT1-MMP expression and glioma expansion. *Neuro Oncol* (2013) 15:1457–1468. doi: 10.1093/NEUONC/NOT115

3. Del Zoppo GJ, Frankowski H, Gu YH, Osada T, Kanazawa M, Milner R, Wang X, Hosomi N, Mabuchi T, Koziol JA. Microglial cell activation is a source of metalloproteinase generation during hemorrhagic transformation. *J Cereb Blood Flow Metab* (2012) 32:919. doi: 10.1038/JCBFM.2012.11

4. Arendt LM, McCready J, Keller PJ, Baker DD, Naber SP, Seewaldt V, Kuperwasser C. Obesity promotes breast cancer by CCL2-mediated macrophage recruitment and angiogenesis. *Cancer Res* (2013) 73:6080–6093. doi: 10.1158/0008-5472.CAN-13-0926
